# A Preliminary Study on the Mechanical Reliability and Regeneration Capability of Artificial Bone Grafts in Oncologic Cases, With and Without Osteosynthesis

**DOI:** 10.3390/jcm9051388

**Published:** 2020-05-08

**Authors:** Michele Boffano, Nicola Ratto, Andrea Conti, Pietro Pellegrino, Laura Rossi, Giuseppe Perale, Raimondo Piana

**Affiliations:** 1 Oncologic Orthopaedic Division, Department of Orthopaedic and Traumatology, Orthopaedic and Trauma Center, Città della Salute e della Scienza, University of Turin, 10126 Turin, Italy; michele.boffano@gmail.com (M.B.); nicolaratto@hotmail.com (N.R.); pelle.pelle@gmail.com (P.P.); raipiana@gmail.com (R.P.); 2 Department of Orthopaedic and Traumatology, University of Turin, 10126 Turin, Italy; 3 Clinical Research Coordinator, Fondazione per la ricerca sui tumori dell’apparato muscoloscheletrico e rari Onlus, 10143, Turin, Italy; laura.rossi.ts@gmail.com; 4 Industrie Biomediche Insubri SA, via Cantonale 67, 6805 Mezzovico-Vira, Switzerland; giuseppe@ibi-sa.com; 5 Faculty of Biomedical Sciences, University of Southern Switzerland (USI), Via G. Buffi 13, 6900 Lugano, Switzerland; 6 Ludwig Boltzmann Institute for Experimental and Clinical Traumatology, Donaueschingenstrasse 13, 1200 Vienna, Austria

**Keywords:** bone grafting, bone regeneration, bone tumor, osteointegration

## Abstract

Several bone grafts are available for clinical use, each with their own peculiar biological and mechanical properties. A new bone graft was obtained by combining mineral structures from natural bovine bones with bioresorbable polymers and cellular nutrients. The study aims to evaluate the clinical, biological and structural properties of this bone graft and its reliability in orthopedic oncology. 23 adult patients (age range 18–85 years) were treated between October 2016 and December 2018; the oncologicdiagnoses were heterogeneous. After surgical curettage and bone grafting, a clinical-radiological follow up was conducted. Radiographs were used to evaluate graft integration according to the usual bone healing and oncologic follow up. Local complications (infection, local recurrence, wound dehiscence, fracture or early reabsorption) were evaluated. The mean followup was of 18.34 ± 4.83 months. No fracture or infection occurred. One case of patellar Giant Cell Tumor (GCT) and one of proximal tibia low-grade chondrosarcoma recurred after about one year. Two wound dehiscences occurred (one required a local flap). Follow-up X-rays showed good to excellent graft integration in most patients (20 out of 21). The investigated graft has a mechanical and structural function that can allow early weight-bearing and avoid a preventive bone fixation (only needed in four patients in this series). The graft blocks are different for shapes and dimensions, but they can be customized by the producer or sawcut by the surgeon in the operating theatre to fit the residual bone cavity. The complication rate was low, and a rapid integration was observed with no inflammatory reaction in the surrounding tissues. Further studies are mandatory to confirm these promising results.

## 1. Introduction

Whether autologous or heterologous, bone grafting is a widely used procedure to fill bone defects and stimulate bone healing in several situations, from traumatic bone defects, delayed union and non-union, to various limb reconstruction techniques. Autologous bone graft has become commonly used over the years as it combines osteogenic, osteoconductive and osteoinductive properties. Indeed, an ideal bone substitute should allow vascular ingrowth and bone remodeling, while stimulating osteoblast progenitors, which configures the autologous iliac bone graft as the current gold standard in bone transplantation [1,2,3]. Although there are advances in harvesting, the procedure is not uncommonly associated with donor-site morbidity such as chronic pain, superficial infection, haematoma and nervous lesions leading to paresthesia and gait alterations [4,5,6]. Moreover, autologous bone graft has limited availability, and it is often not enough when filling large bone defects in orthopedic oncology. On the other hand, allografts are expensive, can potentially cause an immunogenic response by the host and disease transmission, and it is not rare to have bone graft reabsorption [7,8,9]. Allograft is normally preferred when large bone defects (usually larger than 5 cm along the major axis) are encountered [10,11]. Several bone substitutes are available on the market, each with its own biological and biomechanical properties, but rarely fulfilling the ideal bone graft characteristics [12,13,14,15].

**Figure 1:**
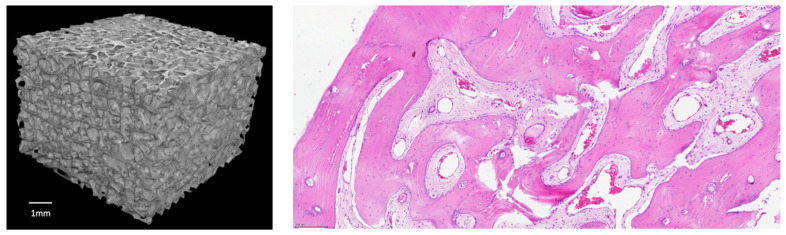
(**left**) 3D reconstruction from a CT scan (Bruker system) on an exemplificative SB block 7 × 7 × 7 mm (reference bar is 1 mm); (**right**) H/E staining on a histologic sample taken after 2.5 years post SB implantation: the graft is completely substituted, and the osteogenesis has formed a lamellar bone with cement lines; a lot of osteocytes inside the lacunae and a good angiogenesis are evidenced (adapted from Grecchi et al. [21]).

Bovine xenografts are valid scaffolds for bone grafting as they mark scores which are very close to human cancellous bone, even if the sterilization process may alter their biomechanical properties [16]. Recently, a low temperature processing of the bone and the addition of composite technology to the xenograft to increase its biological and mechanical performances has been scoring promising results in clinical practice [17,18,19]. SmartBone^®^ (SB) (IndustrieBiomedicheInsubri IBI SA; Mezzovico-Vira, Switzerland) is a composite xeno-hybrid graft that is obtained from a low-temperature processed bovine-derived mineral matrix extracted from the adult bull internal part of femur heads, enriched with the synthetic aliphatic polyester poly(L-lactide-co-ε-caprolactone) (PLCL) and Arg-Gly-Asp (RGD)-containing collagen fragments (obtained from animal-derived gelatin), which altogether improve elasticity, blood affinity and cell attachment, while also providing an open porous and interconnected microenvironment (av. pore size is 250 microns) favorable to cell and vessel colonization, and finally allowing a complete remodeling overtime [18,19,20]. The aminoacidic RGD sequence, alias the tripeptide Arg-Gly-Asp consisting of Arginine, Glycine, and Aspartate, is indeed the preferred motif with the highest affinity for cellular integrins. SB mechanical performances can be compared with human healthy cortico-cancellous bone and are summarized in Table 1 (adapted from [20]). Furthermore, a reconstruction from a CT scan on an SB block and a histologic sample taken after SB implantation can be found in Figure 1.

The present article presents the result of our research hypothesis, i.e., to investigate the biomechanical behavior of the above-mentioned bovine graft in the clinical practice of an oncologic orthopedic setting, further confirming already known biomaterial performances [20], not only from clinical and biologic perspectives but also from a biomechanical point of view: allowing, where possible, to avoid the use of osteosynthesis, hence speeding up patients’ recovery and overall clinical benefits. This work presents the results from a subset of oncologic patients taken from a wider clinical study on SB (see below for details), sharing hence the same endpoints, i.e., the safety and performance of SB in the specific context of such patients. Hence, the objectives of this study, as stated in the approved clinical study protocol, were: (a) to evaluate the overall occurrence of graft failures, the possible risks for local complications such as infection, local recurrence of disease and wound dehiscence; (b) to evaluate graft performances in terms of bone regeneration, assessing the mechanical behavior, and avoiding, where possible, the use of preventive bone fixation; indeed, possible fractures and early reabsorption were investigated, and the osteointegration status was evaluated to assess the ability of the xenograft to integrate with the surrounding bone and to improve new bone deposition and overall remodeling.

## 2. Materials and Methods

All patients, who underwent surgical curettage and SB grafting of a bone lesion in the Orthopaedic Oncologic Department of a national reference center for musculoskeletal tumor surgery from October 2016 to December 2018, were initially retrospectively evaluated. Ethics Committee approval was obtained, and Good Clinical Practice principles were always followed (see details below). Patients under 18 years of age or without a minimum followup of 12 months from surgery were excluded, and 23 patients were finally considered for the study. After being adequately informed about data collection and submission for publication, all patients understood and agreed. The demographic data from patients included in the study and the biomechanical data are presented in Table 2.

The mean age at the time of surgery was 41.9 years old, while the sample presented a female prevalence (9 males and 14 females). Lesions were respectively located: one in the pelvis, eight in the femur (four proximal femur, four distal femur), one in the patella, six in the proximal tibia, five in the proximal humerus (one of them was diagnosed after pathological fracture) and two in the hand (one phalanx and one metacarpal bone). Diagnosis was heterogeneous: low-grade Chondrosarcoma in four cases, Giant Cell Tumor (GCT) in two cases, Enchondroma (four cases), Benign Fibrous Histiocytoma (two cases), Unicameral Bone Cysts (four cases), Aneurismal Bone Cyst (two cases), Fibrous Dysplasia (three cases), Non-Ossifying Fibroma (one case) and Myxofibrosarcoma with bone involvement (one case). The average size of the defect was 62.0 ± 29.8 cm^3^ (range 0.18–111.3), calculated on a preoperative MR exam.

Regarding surgery, the bone lesion was first entirely removed with a Volkmann bone curette through a window made in the intact cortical lamina, opened as a flap. In chondrosarcoma and GCT lesions, high speed burr additional curettage was performed in each surgery with the use of an adjuvant agent (usually alcohol). Whenever feasible, decompression of the proximal and/or distal medullary canal was performed along with the curettage, to improve tumor excision and to enhance healing. The remaining bone defect was filled with the compact positioning of granules and/or duly shaped SB blocks along the direction of major stress forces, depending on the location and size of the bone defect. Afterwards, the cortical lamina was closed back in position. In four cases from our series, a preventive fixation was performed as the defect involved more than half of the circumference of the bone in an axial view. Sutures of the periosteum and surrounding soft tissues completed the surgery.

The patient with bone involvement from myxofibrosarcoma underwent surgical excision of the soft tissue mass and a geometrical cortical resection, followed by a preventive fixation of the humerus and apposition of SB blocks to reconstruct the lateral cortex.

Post-operatively, follow-up and rehabilitation protocols were tailored to each patient, given the typical oncological diversity of each case. Generally, toe-touch weight-bearing with crutches or a walker for the first four to eight weeks was prescribed to patients with a lower-extremity lesion, while for an upper-limb lesion, active elevation of the arm was restricted for the first 15 days and throwing, lifting and strength-training were prohibited for a period of six weeks post-operatively. Patients with upper- and lower-extremity lesions were generally allowed to return to normal activities with no restrictions two and three months post-operatively, respectively. Patients underwent rigorous clinical and radiological follow-up visits, at one month from operation, at 3 months, 6 months and a year from surgery. The clinical examination investigated the state of the wound, the range of motion of the affected joint, possible pain, the radiological appearance of the lesion and overall patient satisfaction. A successful clinical result was considered when the patient presented a normal function, reporting no pain and a satisfying return to normal life. All patients included in the study were evaluated through conventional plain X-rays used to evaluate graft integration and bone healing, while CT or MR were alternatively run during a follow-up visit to rule out possible local recurrences, if oncologically indicated. The primary objective of the study was to evaluate the state of graft integration. This was radiographically graded on a 4-point scale according to Van Hoff [22]: grade 1 (lucency traversing the entire defect space), grade 2 (lucency partly traversing the entire defect space), grade 3 (trabeculae crossing the entire defect space) and grade 4 (defect no longer distinguishable from the surrounding bone). Additionally, the same endpoint was evaluated by means of a radiological protocol specifically designed by Mosetto and colleagues, as described in Table 3 [23]. The score from each radiological exam was collegially evaluated by two orthopedic surgeons (NR, AC). The integration assessment by means of CT scan was taken into consideration, but finally not adopted to avoid excessive irradiation to the patients. Furthermore, any possible local complications such as infection, local recurrence, wound dehiscence, fracture or early reabsorption were investigated during follow-up visits.

Ethics approval and consent to participate: This study is part of an observational study protocol that is sponsored by I.B.I. SA and was approved by the United Ethical Committee of the “Città della Scienza e della Salute”, Turin, Italy (approval n. 0004336), which comprehends also the hospital where all patients were treated. All patients signed an informed consent form to document that they understood the aims of the study and authorized the use of their data for research purposes. Patients were allowed to ask questions pertaining to this study and were thoroughly informed consequently. All procedures were performed in strict accordance with the recommendations of the Declaration of Helsinki as revised in Fortaleza (2013) for investigations with human subjects and followed good clinical practice and ISO14155 prescriptions.

## 3. Results

The population presented a median (IQR) age at the time of surgery of 41 (26:53.5) years, with 61% of the patients enrolled for the study represented by females, while only 39% were males. At the last timepoint, at the publication date, the mean followup was of 20.34 ±4.83 months (range: 12–31 months). In the post-operative period, two wound dehiscences occurred: one of them healed by secondary intention, while the other one required a local flap. No deep infections or other early complications occurred in the same period.

Of the 23 patients, two cases of local recurrence occurred after one year: one case of patellar GCT and one of proximal tibia low-grade chondrosarcoma. The patient with recurrent patellar GCT underwent a wide resection of the extensor apparatus and reconstruction with a transplant from a cadaveric donor. The huge chondrosarcoma lesion relapsing in the surrounding soft tissues was biopsied again and resulted in a dedifferentiated chondrosarcoma, thus the patient underwent above-knee amputation. Except for the above-mentioned patients, the rest experienced no pain at the last followup. Only one patient reported a slight pain referable to the proximal medial tibial metal plate that was implanted to increase primary stability. 

At the last follow-up visit, all surgical scars were neatly healed, and the clinical range of motion was comparable to a normal joint in the whole sample of patients, except for the patient with metacarpal enchondroma, who experienced a residual limited flexion of the same finger which, however, did not affect her daily and working life. Generally, the patients reported an overall satisfaction with the surgery. No fractures or infections were detected.

Plain radiographs at the last followup were used to evaluate the osteointegration with the bone substitutes, and the results obtained were reported in Table 4. The above-mentioned recurrent lesions were excluded in the radiological assessment of the integration state as these were not evaluable due to the presence of pathological tissue.

## 4. Discussion

The surgical procedure of curettage is indicated for the treatment of benign, borderline and some low-grade malignant bone tumors, such as GCT or grade I chondrosarcoma. The resultant bone defect is usually filled with bone graft or synthetic bone with the aim of promoting bone growth into the residual cavity. Besides not being immunogenic, the perfect substitute should ideally possess the same biomechanical characteristics as the native bone. It should hold growth factors and cytokines to recruit stromal stem cells and stimulate their development into osteoblast progenitors (osteoinduction), and serve as a scaffold for new bone cells and capillary colonization (osteoconduction) to finally allow bone ingrowth (osteogenesis) [24,25,26].

Over the last years, an increasing number of bone substitute materials were introduced on the market as an alternative to autologous and allogenic grafts [27,28,29,30,31]. Some pure advantages of synthetic bone grafts are evident, such as no additional donor-site morbidity, no risk for disease transmission, less operating time and unlimited availability. Even if most of them only have osteoconductive properties and variable (high to none) osteoinductive properties, literature data are promising. A recent systematic review demonstrated comparable healing after a surgical curettage for benign bone lesions when using either an autograft/allograft or a bone substitute. The resulting healing rate was approximately around 90% [32]. Similar findings were reported between two groups of patients treated with the curettage of a unicameral bone cyst. A complete healing was achieved in 64.3% in the bone substitute group and 57.1% in the allograft group [33]. Additionally, the type of bone graft or substitute was proved not to affect the outcome in instrumented spine fusion for adolescent idiopathic scoliosis [34]. More recently, some authors have focused their studies on xeno-hybrid materials such as SB, which combines the biological qualities of auto- and allografts along with the mechanical performances of synthetic scaffolds [35]. It has been proven that its affinity to blood facilitates attachment by Mesenchymal Stem Cells (MSCs), which then differentiate in osteoblasts, leading to the deposition of a new bone matrix that eventually degrades and substitutes the SB [19]. SB has already been safely used in both oral and maxillofacial surgery, and in orthopedic trauma for the treatment of tibial plateau fractures with promising results [18,19,36]. This graft is available in a wide variety of shapes and dimensions and is also customizable by the producer in advance or can be sawcut directly by the surgeon in the operating theatre to fit the residual gap [20].

**Figure 2:**
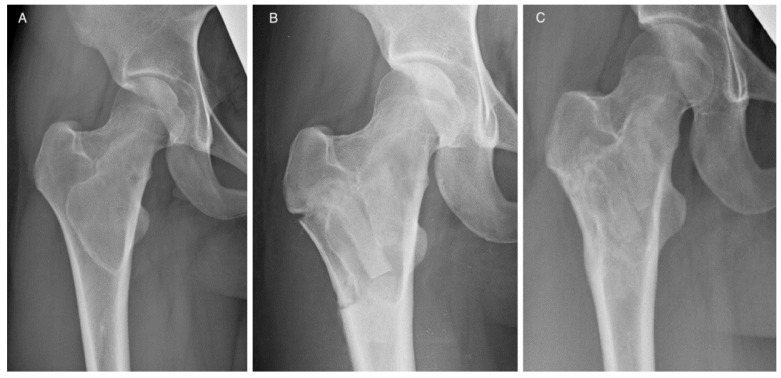
Patient 19. (**A**) Proximal right femur Fibrous Dysplasia. Radiological evolution after curettage and SB grafting with shaped blocks after (**B**) 3 and (**C**) 6 months of follow up.

**Figure 3:**
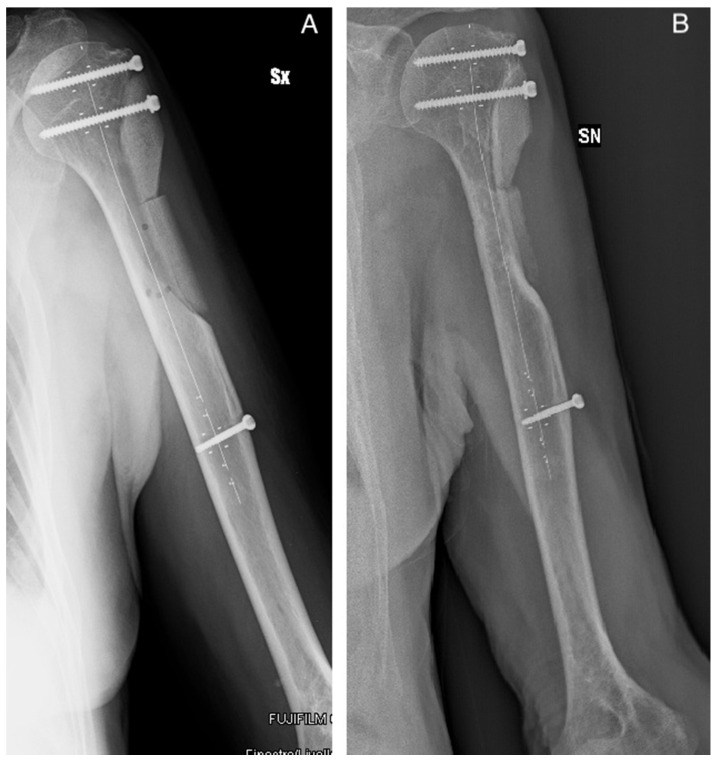
Patient 18. Radiological control after one month from Myxofibrosarcoma excision with (**A**) lateral proximal humerus cortical resection and reconstruction with two SB blocks. (**B**) The radiograph on the right shows a line of radiolucency and a partial resorption of the lowest block due to the incomplete coverage of the graft and its isolation from the soft tissues. Abbreviations: SX and SN stand for Left.

The handling and workability of this biomaterial and its mechanical properties were known, as they were previously investigated in vitro [20,37] and assessed in clinical applications [19,36,37]. Moreover, complex cases were also investigated in silico [38] in order to determine biomaterial limits under extreme loads. These evidences, together with the analysis of each case here reported (see Table 2), allowed for early weight-bearing and avoiding a preventive bone fixation in most of the patients. Only in four cases was bone stabilization necessary because for three of them more than two thirds of the cortical circumference of the tibia or femur were involved, in patients with a relevant Body Mass Index. In the other case, an extensive cortical resection was performed. In some specific cases, the positioning of SB blocks longitudinally along the major mechanical axis of the bone may give the opportunity to obtain higher stability [38]. The radiological evaluation of the graft integration was good or excellent according to Mosetto’s six-grade score in 95% of the sample (20 out of 21 patients). Figure 2 shows the progressive optimal integration of SB after curettage of a proximal femur Fibrous Dysplasia. The poor result was in the patient with myxofibrosarcoma, where the SB blocks were used to reconstruct the lateral cortex of the humerus. In this case, the radiolucent line surrounded the whole graft, which was also partially reabsorbed (Figure 3). At the same time, no major complications occurred. The risk for recurrence is related to the characteristics of the disease and the performed curettage. A more aggressive curettage, if indicated, can also increase local bleeding and help bone healing and graft integration. This product appeared valid and safe not only in benign bone diseases. The case of recurrence occurring to the patient with patellar GCT initially presented an interruption of the anterior patellar cortex and an involvement of the surrounding soft tissues. An inadequate lesion removal may have caused an early relapse and progression of the disease, but as seen on the MR scan and intraoperatively, an important disease dissemination occurred in the host tissue. On the contrary, the SB block previously implanted was still intact and was not invaded by pathological tissue. Such preliminary results may suggest that SB does not help vehiculate the disease.

Several important limitations affect this study. Mainly, this is a small cohort retrospective study, and only cases treated in the past with this specific product have been included, so that the selection method is a subjective rather than an objective one. Furthermore, diagnoses were heterogeneous and included benign and low-grade malignant lesions with different biological behaviors, so that the comparison of the bone healing of the lesions might not be completely accurate. Furthermore, the radiological integration status of the bone substitute may have been more accurately calculated with serial CT scans at follow-up visits because different trabecular patterns could be detectable, but the patients were spared unnecessary irradiation [39]. This was taken into consideration and, for this reason, finally not adopted. Again, this xenograft substitute was not compared with any other materials, even if complete bone healing after curettage might not be correlated to the nature of the filling material used but to the quality of curettage [40]. Glancy et al. reported a higher “no healing” rate for tumors with a large size after curettage and grafting for bone tumors [41].

## 5. Conclusions

SB results in a safer and valid bone graft substitute: it has a mechanical structural function that can allow early weight-bearing in selected cases and avoid preventive bone fixation devices, hence sparing second removal surgeries where potentially needed. Cortical bone may be interrupted or resected, but if a graft with similar biomechanical characteristics is used the constructresidual bone-graft can be considered homogeneous and with a low risk of fracture. The complication rate was low, and a rapid integration was observed with no inflammatory reaction in the surrounding tissues. The graft is customizable by the producer or directly by the surgeon in the operating theatre and is available in different shapes and dimensions. The collected data overall confirms biomaterial clinical performances. In view of the specificity of the study’s main addressed aims, and given the relatively small patient size, further investigations are suggested to confirm these promising results.

## Figures and Tables

**Table 1:** SmartBone^®^ mechanical properties (adapted from [20]).

**Torsion**	**Max Torque (Nmm)**	**Max Stress (MPa)**	**Max Strain %**	**Torsional Elastic Modulus (MPa)**	**Kg/cm^2^**
medium value	1504.4	25.5	5.8	490.6	259.8
standard deviation	294.9	4.4	0.9	103.7	44.9
**Bending**	**Max Force (N)**	**Max Stress (MPa)**	**Max Strain %**	**Flexural Modulus (MPa)**	**Kg/cm^2^**
medium value	100.3	23.8	7.6	340.6	242.4
standard deviation	17.4	4.2	0.9	63.1	42.4
**Compression**	**Max Force (N)**	**Max Stress (MPa)**	**Max Strain %**	**Elasticity Modulus (MPa)**	**Kg/cm^2^**
medium value	1914.2	25.8	2.2	1245.7	262.9
standard deviation	590.6	7.8	0.4	225.9	80.1

**Table 2:** Demographic data from patients included in the study. The seventh column refers to the percentage of bone involved in an MR or CT axial view. The following legend was applied: 0 points if intramedullary, 1 point when occupying less than ⅓ of bone diameter, 2 points if between ⅓ and ⅔ of bone diameter, and 3 points when involving more than ⅔ of bone diameter.

	Age	Sex	Diagnosis	Location	Defect Size (cm^3^)	Cortical Bone Involved (%)	Osteosynthesis (yes/no)	Height (cm) × Body Weight (kg)
1	29	F	UBC	Proximal Femur	4.3 × 3.3 × 3 = 42.6	1	no	165 × 50
2	58	M	FD	Proximal Tibia	2.5 × 1.8 × 3.1 = 13.9	1	no	176 × 84
3	41	M	Grade I CS	Hand	0.5 × 0.9 × 0.4 = 0.18	2	no	173 × 75
4	42	F	Enchondroma	Distal Femur	8.6 × 1.1 × 0.8 = 7.5	2	no	154 × 51
5	43	F	Grade I CS	Proximal Tibia	5.7 × 4.6 × 3.8 = 99.6	1	no	166 × 74
6	18	M	NOF	Proximal Tibia	4.4 × 2.4 × 2.8 = 29.5	1	no	183 × 62
7	37	F	Enchondroma	Hand	2.9 × 0.7 × 0.7 = 1.4	2	no	175 × 50
8	39	F	Enchondroma	Proximal Humerus	4.2 × 2.6 × 2.4 = 26.2	1	no	158 × 56
9	58	F	FD	Proximal Tibia	8.1 × 2.7 × 2.9 = 63.4	1	no	160 × 75
10	57	F	UBC	Pelvis	5.7 × 3.8 × 3.3 = 71.5	2	no	168 × 58
11	51	F	UBC	Proximal Humerus	4.2 × 1.4 × 3.1 = 18.2	2	no	165 × 66
12	20	M	UBC	Proximal Humerus	4.8 × 2.9 × 2.5 = 34.8	1	no	175 × 60
13	31	F	ABC	Distal Femur	5.1 × 3.8 × 3.2 = 62	2	no	155 × 51
14	23	M	BFH	Distal Femur	10.3 × 1.3 × 1.1 = 14.7	3	yes	176 × 78
15	44	F	Grade I CS	Proximal femur	5.3 × 1.4 × 1.3 = 9.6	1	no	156 × 55
16	18	F	BFH	Distal Femur	11.1 × 3.2 × 1.4 = 49.7	2	no	160 × 50
17	65	F	Enchondroma	Proximal Tibia	4.0 × 2.2 × 2.7 = 23.7	1	no	155 × 68
18	84	M	MXFS with bone involvement	Proximal Humerus	9.0 × 1.1 × 2.1 = 20.8	3	yes	180 × 78
19	18	F	FD	Proximal Femur	6.0 × 3.1 × 2.3 = 42.8	1	no	163 × 58
20	40	M	ABC	Distal Femur	4.4 × 4.6 × 5.5 = 111.3	3	yes	167 × 74
21	51	F	Grade I CS	Proximal Humerus	6.4 × 1.5 × 0.9 = 8.6	1	no	162 × 67
22	18	M	GCT	Patella	3.4 × 1.5 × 3.7 = 18.9	2	no	180 × 70
23	56	M	GCT	Proximal Tibia	2.7 × 3.3 × 3.1 = 27.6	3	yes	165 × 55

**Table 3:** Radiological follow-up form to value autografts and allografts, proposed by Mosetto [23].

X-Rays in 2P	Points	Result
Graft reabsorption		
Total	−2	
Partial	−1	Poor—Consider graft removal
No change in graft radiodensity from post-operatory radiographs	0	Poor—Consider graft removal
Increase in graft radiodensity without integration with patient’s bone (radiolucent line still visible)	+1	Fair—Wait and see
Increase in graft radiodensity with integration with patient’s bone		
Partial	+2	Good—Wait and see
Total	+3	Excellent

**Table 4:** Report on the evaluation of the state of graft integration at the last follow up according to Van Hoff and Mosetto radiological scores [22,23].

Van Hoff Score	Number of Patients	Mosetto Score	Number of Patients
1	1	−2	0
2	0	−1	1
3	14	0	0
4	6	+1	0
		+2	8
		+3	12

## Abbreviations

F	Female
M	Male
UBC	Unicameral Bone Cyst
FD	Fibrous Dysplasia
CS	Chondrosarcoma
NOF	Non-Ossifying Fibroma
ABC	Aneurismal Bone Cyst
BFH	Benign Fibrous Histiocytoma
MXFS	Myxofibrosarcoma
GCT	Giant Cell Tumor
